# Investigating Neuropharmacological Features of the Cortical Activity of Cannabidiol GWP42003 P—A Phase 1 Clinical Trial

**DOI:** 10.3390/brainsci16090957

**Published:** 2026-09-09

**Authors:** Viviana Santoro, Po-Yu Fong, Andrea Biondi, Isabella Premoli, Harry Clark, Lorenzo Rocchi, Mark P. Richardson

**Affiliations:** 1 Department of Basic and Clinical Neuroscience, Institute of Psychiatry Psychology and Neuroscience (IoPPN), King’s College London, London WC2R 2LS, UK; viviana.santoro@kcl.ac.uk (V.S.); andrea.2.biondi@kcl.ac.uk (A.B.); isabella.premoli@saniona.com (I.P.); 2018899c@student.gla.ac.uk (H.C.); mark.richardson@kcl.ac.uk (M.P.R.); 2 Headache Group, Wolfson SPaRC, Institute of Psychiatry Psychology and Neuroscience (IoPPN), King’s College London, London WC2R 2LS, UK; neurologist.fong@gmail.com; 3 Division of Movement Disorders, Department of Neurology and Neuroscience Research Centre, Chang Gung Memorial Hospital at Linkou, Taoyuan City 33305, Taiwan; 4 Medical School, College of Medicine, Chang Gung University, Taoyuan City 33302, Taiwan; 5 Department of Clinical and Movement Neurosciences, UCL Queen Square Institute of Neurology, University College London, London WC1E 6BT, UK; 6 Saniona A/S, Murervangen 42, 2600 Albertslund, Denmark; 7 Centre for Cognitive Neuroimaging, School of Psychology and Neuroscience, University of Glasgow, Glasgow G12 8QQ, UK; 8 Department of Medical Sciences and Public Health, University of Cagliari, 09124 Cagliari, Italy

**Keywords:** cannabidiol, transcranial magnetic stimulation, electroencephalography, TMS-EEG, cortical excitability, epilepsy, neural synchronization, electromyography, GABA receptors, anti-seizure medications

## Abstract

**Highlights:**

**What are the main findings?**

- A dose of purified cannabidiol did not significantly alter cortical excitability or established GABAergic biomarkers.
- The drug exhibited a trend toward increased alpha and decreased delta neural synchronization compared to placebo.

**What are the implications of the main findings?**

- The absence of significant effects dictates a cautious interpretation of potential GABAergic modulation.
- Capturing the distinct neuropharmacological mechanisms of cannabidiol on cortical networks may require chronic dosing rather than acute administration.

**Abstract:**

**Background/Objectives**: Despite the clinical efficacy of cannabidiol (CBD) in treating certain epilepsies, its in vivo neuropharmacological mechanisms remain incompletely understood. This Phase 1 clinical trial aimed to evaluate the acute effects of a single oral dose of highly purified CBD (GWP42003-P) on cortical excitability. Transcranial magnetic stimulation combined with electroencephalography (TMS-EEG) and electromyography (TMS-EMG) were utilized as assessment tools. **Methods**: In a randomized, double-blind, placebo-controlled crossover trial, 15 healthy male participants received a single 1500 mg dose of GWP42003-P or placebo. Cortical activity metrics, including resting and active motor thresholds (RMT, AMT), short intracortical inhibition (SICI), TMS-evoked potentials (TEPs), TMS-related spectral perturbations (TRSP), and inter-trial phase clustering (ITPC), were recorded pre-dose and at 1, 4, and 6 h post-dose. **Results**: RMT and AMT significantly decreased over time following CBD administration, though without a significant Condition × Time interaction. While primary analyses showed no condition-driven alterations for SICI, TEPs, or TRSP, secondary longitudinal modeling revealed a transient reduction in beta-band desynchronization 1 h post-dose, aligning with peak plasma concentration. The drug condition also exhibited a qualitative, non-significant trend toward increased alpha ITPC and decreased delta ITPC at 4 and 6 h post-dose compared to placebo. **Conclusions**: A single acute 1500 mg dose of GWP42003-P did not produce statistically significant alterations in widespread cortical excitability in healthy adult males. However, the transient blunting of beta desynchronization at peak concentration may reflect CBD’s distinct, non-classical neuromodulatory profile. Overall, these largely null findings suggest that acute CBD administration lacks a robust, direct modulatory effect on cortical networks. Capturing its precise neuropharmacological mechanisms will likely require future investigations utilizing chronic dosing paradigms or clinical patient populations.

## 1. Introduction

Epilepsy comprises a group of disorders characterized by recurrent, unprovoked seizures. As one of the most common neurological conditions, it encompasses numerous causes, risk factors, and clinical presentations, all reflecting an underlying chronic neural dysfunction [1]. While anti-seizure medications (ASMs) provide satisfactory seizure control for most patients, approximately 30% are non-responsive to treatment [2], for reasons that remain only partially understood.

Among novel therapeutics, highly purified cannabidiol (CBD; GWP42003-P) has demonstrated anti-seizure efficacy in clinical trials for childhood-onset epilepsies; however, the precise mechanisms underlying these effects in humans remain elusive. Preclinical evidence [3,4,5,6] suggests that CBD mitigates neuronal hyperexcitability through three primary pathways: (i) modulating intracellular calcium by antagonizing G protein-coupled receptor 55 (GPR55) [7]; (ii) regulating extracellular calcium influx via transient receptor potential vanilloid type 1 (TRPV1); and (iii) inhibiting adenosine transport [3,5,8].

Because most ASMs modulate receptors and ion channels critical for regulating neuronal electrophysiology, developing methods to assess in vivo cortical excitability is essential for evaluating the pharmacological mechanisms of agents like CBD. To this end, transcranial magnetic stimulation (TMS) is frequently combined with electromyography (EMG) and electroencephalography (EEG) to investigate central nervous system (CNS) drugs with well-established mechanisms of action (e.g., those targeting specific receptors like GABAAR or GABABR). In TMS-EMG studies, the resting motor threshold (RMT)—defined as the minimum stimulation intensity required to elicit a motor evoked potential (MEP) in 50% of trials [9]—serves as a biomarker of net axonal excitability and is particularly sensitive to voltage-gated sodium channel blockade [10,11]. Short intracortical inhibition (SICI), measured by the suppression of MEPs following a preceding subthreshold stimulus, provides another valuable metric, as it reflects short-lasting postsynaptic inhibition mediated via GABAAR [12,13].

Although TMS-EMG effectively evaluates corticospinal excitability following primary motor cortex (M1) stimulation, the recorded muscle responses inherently involve subcortical, spinal, and peripheral nerve pathways [14]. In contrast, combining TMS with EEG offers the advantage of recording evoked responses directly at the cortical level across both motor and non-motor areas. Following a single TMS pulse, these EEG responses emerge as a summation of postsynaptic potentials, appearing as a series of positive and negative deflections at specific latencies known as TMS-evoked potentials (TEPs). These responses propagate from the stimulation site to other brain regions and persist for approximately 300 milliseconds [15,16]. Centrally acting drugs can significantly alter TEP morphology. For example, inhibition mediated by synaptic alpha-1 (phasic) and extra-synaptic alpha-5 (tonic) GABAAR subunits contributes to responses at 45 msec (N45), whereas responses at 100 msec (N100) are associated with GABAB receptor-mediated inhibition [17]. Furthermore, the amplitude of peaks at 25/30, 45, and 180 msec is modulated by anti-seizure medications targeting voltage-gated sodium channels, such as lamotrigine and carbamazepine, as well as those targeting synaptic vesicle glycoprotein 2A, such as levetiracetam [18,19]. Similarly, TMS-EEG was also utilized in a Phase I clinical trial to characterize XEN1101, a novel positive allosteric modulator (or “opener”) of the KCNQ2/3 (Kv7.2/7.3) potassium channel. A single dose of XEN1101 effectively suppressed TEP responses at 30, 45, and 180 msec post-stimulation [20]. Furthermore, beyond these standard offline pharmacological assessments, emerging paradigms are increasingly leveraging real-time EEG readouts to characterize ongoing cortical states and inform adaptive, closed-loop neuromodulation strategies [21].

Beyond the time domain, TMS-EEG responses can be analyzed in the time-frequency domain. Unlike TEPs, this approach captures information that is not strictly phase-locked to the stimulus. These broad-frequency changes in spectral power over time are known as TMS-related spectral perturbations (TRSP) and encompass both phase-locked and non-phase-locked EEG activity [22,23,24]. Following M1 stimulation, TRSP exhibits a characteristic pattern: an early increase in delta, theta, alpha, and beta band power up to 200 msec, followed by alpha and beta suppression (often termed desynchronization), and concluding with a late increase in beta power around 500 msec [25]. Recent research into the effects of ASMs on TRSP revealed distinct drug-specific profiles: levetiracetam suppressed theta, alpha, and beta power; lamotrigine decreased delta and theta, but increased alpha power; and XEN1101 broadly reduced delta, theta, alpha, and beta power [26].

To date, investigations into the neuropharmacological effects of cannabinoids on human cortical excitability using TMS have revealed compound-specific profiles. For instance, acute administration of delta-9-tetrahydrocannabinol (THC) has been shown to alter cortical excitability, often manifesting as reduced short-interval intracortical inhibition (SICI) or facilitated cortical networks, reflecting its partial agonist activity at CB1 receptors [27]. In contrast to THC, recent evidence suggests CBD does not drastically alter baseline excitability. A recent randomized crossover trial by Gorbenko and colleagues [28] demonstrated that acute administration of CBD lacks a direct, widespread effect on basic cortical excitability and standard TMS-evoked potentials (TEPs) in healthy volunteers.

Building upon these foundational findings, this Phase 1 clinical trial aimed to evaluate the acute effects of a single 1500 mg dose of CBD (GWP42003-P) on cortical activity. We hypothesized that characterizing the in vivo alterations of cortical excitability—by exploring not only motor thresholds and TEPs but also time-frequency dynamics such as TRSP and inter-trial phase clustering (ITPC)—would help elucidate the precise mechanisms underlying CBD’s clinical efficacy, and we investigated whether the administration of GWP42003-P would manifest as measurable changes across these parameters.

## 2. Materials and Methods

### 2.1. Participants

Eighteen healthy, right-handed male participants (mean age: 29.11 ± 6.60 years) were recruited from a local research volunteer database. To control for the influence of body composition on drug distribution, inclusion was restricted to individuals with a body weight ≥50 kg and a Body Mass Index (BMI) between 18.50 and 30.00 kg/m^2^. Right-handedness was confirmed by an Edinburgh Handedness Inventory laterality score of ≥40% [29,30]. Female participants were excluded from the study because the menstrual cycle has been shown to influence cortical excitability [31].

Participants had no personal or family history of neurological or psychiatric disorders and were not taking any medications acting on the central nervous system. All individuals provided written informed consent following an initial screening (Visit 1). Eligibility was strictly determined during this baseline screening visit (conducted between 28 and 1 days prior to the first dosing day). This comprehensive evaluation was not solely reliant on self-report; it included physician-led physical and neurological examinations, 12-lead electrocardiograms (ECGs), serological testing, and extensive clinical laboratory assessments encompassing hematology, clinical chemistry, coagulation, and urinalysis. Participants also underwent the Magnetic Stimulation Adult Safety Screen (MSASS) and the Columbia-Suicide Severity Rating Scale (C-SSRS).

To control for potential lifestyle confounders, participants were instructed to abstain from alcohol and tobacco for two weeks prior to testing. Furthermore, consumption of grapefruit, Seville oranges, pomelos, star fruit, and cranberries was prohibited for seven days prior to the study. Compliance was objectively verified at the initial screening and immediately upon admission for each dosing visit via breath alcohol tests and comprehensive urine drug screens. These urine panels explicitly tested for tetrahydrocannabinol (THC) and other cannabinoids to rule out recreational marijuana use, as well as amphetamines, barbiturates, benzodiazepines, cocaine metabolites, methadone, opiates, phencyclidine, tricyclic antidepressants, and cotinine.

Of the eighteen enrolled participants, one withdrew following the first visit. Two additional participants were excluded after testing positive for cotinine at their second dosing visit, which corresponded to their placebo session. Because their active drug session occurred during their first visit when all drug screens were negative, their pharmacokinetic data were not confounded by nicotine and are included in the GWP42003-P serum concentrations summarized in Table 1. The remaining fifteen participants completed the full experimental protocol. The drug was detectable in the serum of all participants from four hours post-dose onward. No serious adverse events (SAEs) were reported during the study.

All study procedures were approved by the UK Medicines and Healthcare products Regulatory Agency and the London-Brent NHS Research Ethics Committee (reference 20/LO/0327). The study was conducted in accordance with the Declaration of Helsinki and complied with international safety guidelines for non-invasive brain stimulation [32].

### 2.2. Experimental Design

Using a randomized, double-blind, placebo-controlled, two-period crossover design, this study investigated the acute effects of a single oral dose of GWP42003-P versus placebo on various TMS-EMG and TMS-EEG metrics (Registration number: EudraCT: 2019-004717-13; name of the registry: European Union Drug Regulating Authorities Clinical Trials Database; registration date: 20 April 2020). GWP42003-P was administered as a 15 mL oral solution (100 mg/mL), providing a total dose of 1500 mg. The investigational product and the matching placebo were manufactured and supplied by the trial sponsor (Jazz Pharmaceuticals, Dublin, Ireland). The active solution consisted of highly purified CBD (≥98% purity) extracted from *Cannabis sativa* L., formulated in sesame oil with anhydrous ethanol, sweetener, and strawberry flavoring. The placebo was an oral solution matched in appearance and formulation, containing β-carotene instead of CBD. The study medications were identically packaged and labeled by the sponsor, and were securely managed and dispensed by the clinical trials pharmacy at King’s College Hospital. Participants were assigned to treatment sequences based on a computer-generated randomization schedule prepared by an independent statistician. Allocation concealment was strictly maintained throughout the trial; investigators, clinical staff, and participants remained fully blinded, as the active and placebo solutions were matched in appearance, viscosity, and flavoring, and were provided in identical containers.

**Figure 1:**
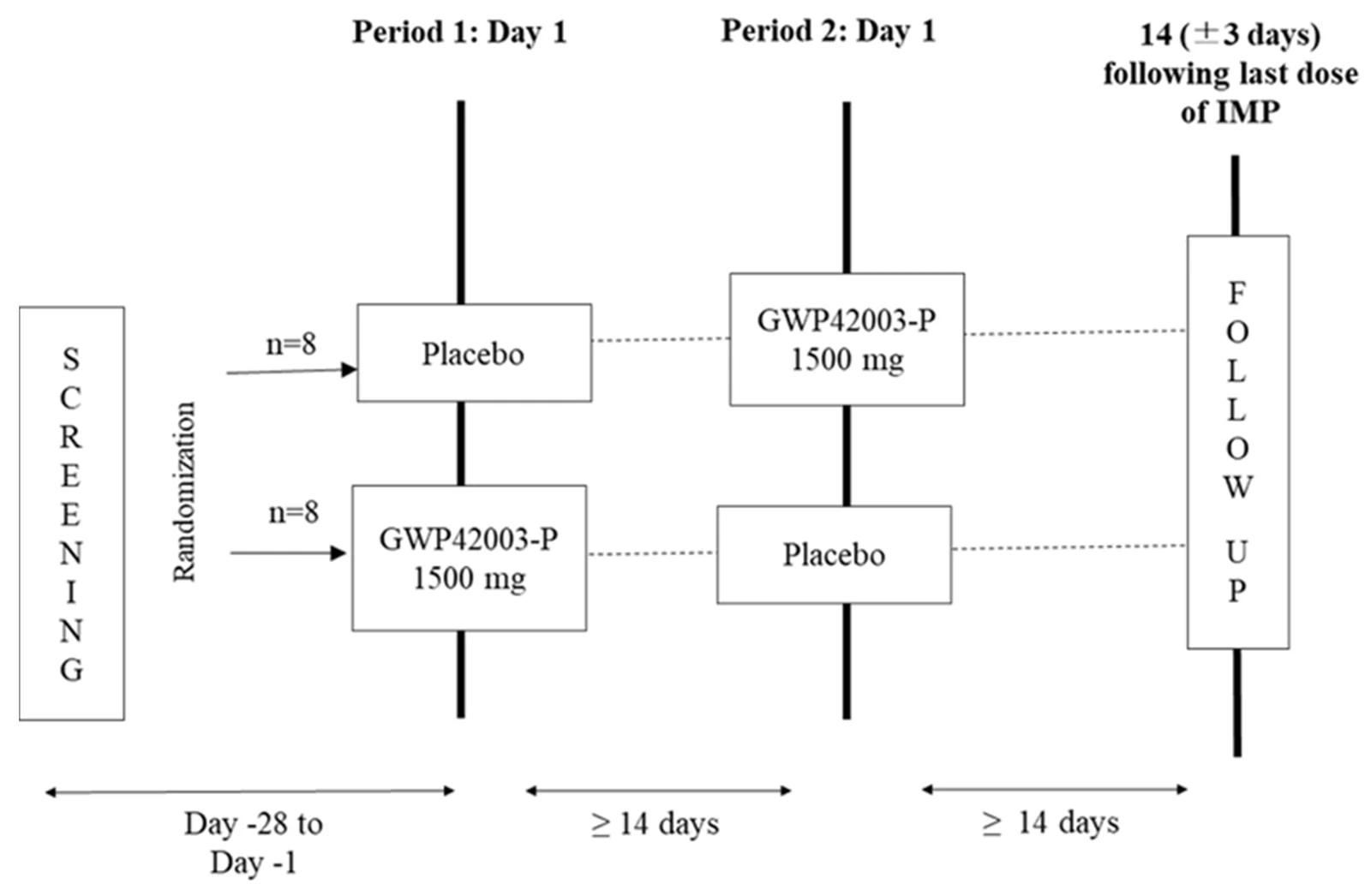
Trial design. Subjects who passed the screening period (Visit 1) were randomized to 1 of 2 possible treatment sequences, and GWP42003-P or placebo was administered in a counterbalanced order. Following a 14-day washout, subjects returned to receive the alternate treatment and returned approximately 14 days later for a follow-up safety visit.

**Figure 2:**
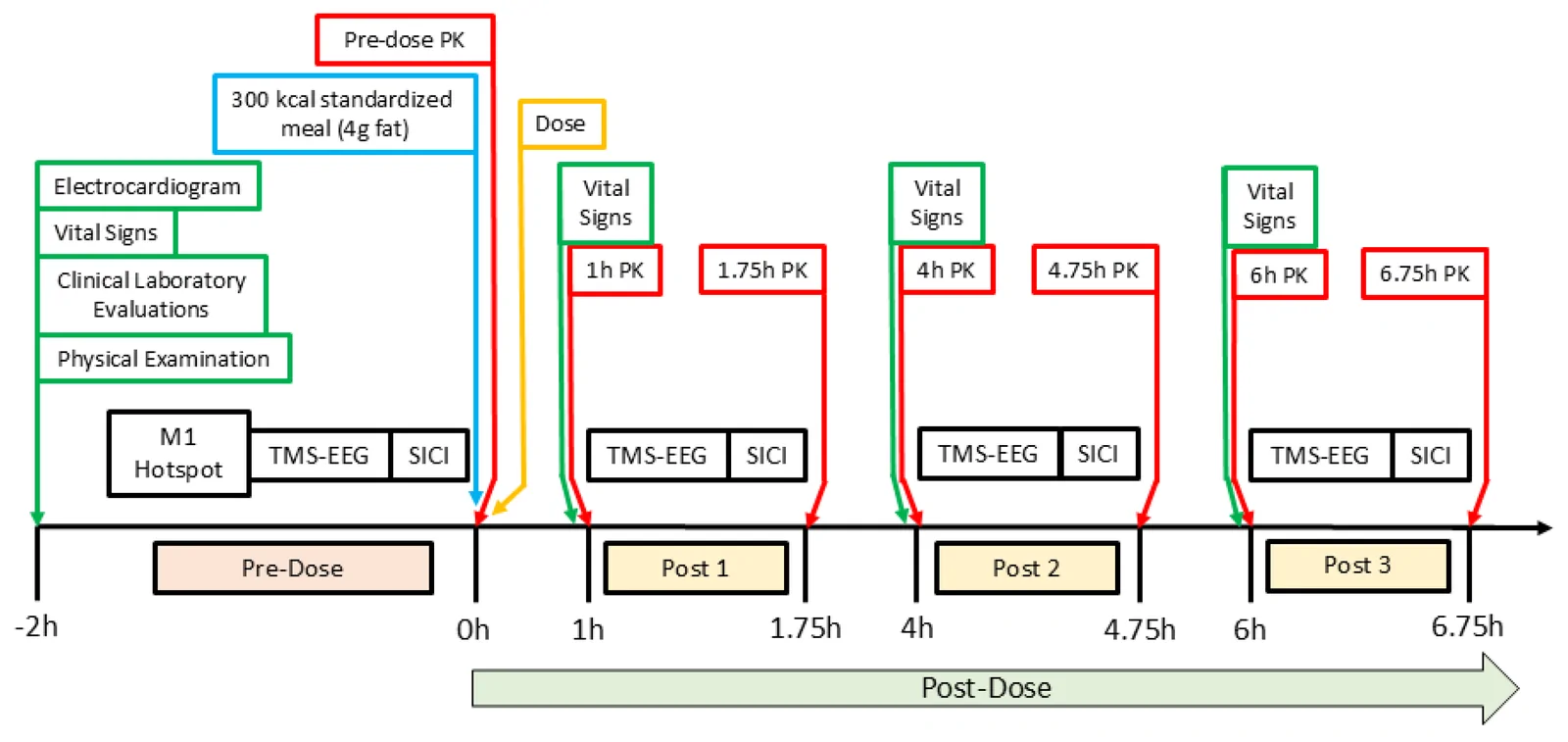
Timing of variable collection. On each dosing day, subjects entered the clinic and completed safety assessments including vital signs, physical examination, electrocardiograms, and clinical laboratory evaluations. On dosing days, subjects were asked to fast from midnight until entering the clinic. They were provided a snack and, once baseline assessments were completed, a standardized meal containing approximately 300 kcal (of which approximately 4 g is composed of fat). TMS assessment was performed at pre-dose and repeated 1 (Post 1), 4 (Post 2), and 6 (Post 3) hours after drug or placebo intake.

All experimental sessions were conducted at the NIHR-Wellcome Clinical Research Facility at King’s College Hospital NHS Foundation Trust (Figure 1). The experimental timeline is illustrated in Figure 2. Participants were required to fast from midnight before entering the clinic. Participants were admitted to the clinical research facility at 9:00 a.m. on each dosing day, and baseline assessments commenced at the same time for all subjects to minimize circadian bias. Upon admission, and approximately two hours prior to dosing, participants underwent a pre-dose clinical examination to confirm continued eligibility; this included a symptom-directed physical and neurological examination, a 12-lead ECG, repeated clinical laboratory safety evaluations, and comprehensive drug, cotinine, and breath alcohol screens. Following these medical checks, the extensive neurophysiological setup—including EEG cap placement and M1 hotspot identification—served to familiarize participants with the experimental procedures and minimize measurement variability prior to baseline data collection.

Following safety and neurophysiological assessments, participants were provided a standardized meal containing approximately 300 kcal (of which approximately 4 g was fat) 30 min prior to dosing. The drug was administered under the supervision of site staff at approximately 11:00 a.m. This specific meal composition was selected because food intake—particularly fat content—increases the rate and extent of CBD absorption and limits inter-individual pharmacokinetic variability.

On each dosing day, TMS-EMG and TMS-EEG data were acquired before drug administration (Pre-Dose) and at 1 (Post 1), 4 (Post 2), and 6 (Post 3) hours post-dose. These intervals were designed to capture the ascending, peak, and post-peak phases of plasma CBD concentrations, as the time to maximum plasma concentration typically occurs at approximately 3 h post-dose. To evaluate pharmacokinetics (PK), serum drug concentrations were measured at each time point, and the peak serum concentration (Cmax) was recorded. During each experimental block, participants were seated in a comfortable reclining chair with their eyes open. To minimize eye movement artifacts, they were instructed to maintain visual fixation on a white cross displayed on the wall.

### 2.3. TMS-EMG Recording and Analysis

EMG was recorded from the right FDI muscle using Ag/AgCl electrodes in a belly tendon montage. Signals were filtered (5–1000 Hz), digitized at 3000 Hz (MEP module, Rogue Research Inc., Montreal, QC, Canada), and analyzed offline (Brainsight software 2.4.5, Rogue Research Inc.). TMS was delivered via a DuoMAG MP-Dual Paired Pulse stimulator connected to a 70 mm figure-of-eight coil (DuoMAG 70BF, Deymed, Czech Republic). We first identified the motor hotspot, defined as the site over the left M1 that consistently elicited the largest peak-to-peak MEP amplitude in the resting contralateral FDI muscle [9,33]. To identify this site, the TMS coil was initially positioned approximately 7 cm laterally from the vertex and held tangentially to the scalp at an approximate 45° angle to the mid-sagittal plane to induce a posterior-to-anterior current. The coil was then systematically moved in small increments while delivering suprathreshold single pulses to map the optimal cortical representation. This defined M1 hotspot was subsequently used for all TMS-EMG and TMS-EEG acquisitions. To ensure consistent targeting and minimize coil drift throughout the experiment, the hotspot and coil positions were continuously monitored using a 3D optical neuronavigation system (Brainsight TMS Navigation, Rogue Research, Canada) [34,35]. We then determined the RMT and AMT. RMT was defined as the minimum stimulation intensity required to evoke an MEP of at least 50 µV in five out of ten consecutive trials in the resting muscle. This metric was measured at each recording time point (RMT_Pre-dose_, RMT_Post1_, RMT_Post2_, and RMT_Post3_). AMT was defined as the lowest intensity capable of eliciting an MEP of at least 200 µV in five out of ten trials while the participant maintained a 10–15% voluntary contraction of the target muscle [9,36]. To investigate the potential effects of GWP42003-P on inhibitory intracortical circuitry, SICI was assessed using a conditioning stimulus at 80% of RMT_Pre-dose_, followed by a test stimulus (TS) at 120% of the RMT corresponding to that specific time point (i.e., RMT_Post1_, RMT_Post2_, or RMT_Post3_), with an interstimulus interval of 2 ms [37,38,39]. A total of 15 conditioned and 15 unconditioned trials were delivered in a pseudorandomized order, with an inter-trial interval of 4 s ± 20% [40,41].

During offline analysis, MEPs were excluded if their amplitude fell outside 1.5 times the interquartile range (i.e., below the first quartile or above the third quartile), or if ongoing prestimulus EMG activity exceeded 50 µV within the 100 ms preceding the TMS pulse) [37,42,43]. Finally, SICI was calculated for each subject as the ratio of the average conditioned MEP amplitude to the average unconditioned MEP amplitude.

### 2.4. TMS-EEG Recording and Analysis

EEG signals were acquired using 64 Ag/AgCl passive electrodes (EasyCap 64Ch; Brain Products, Gilching, Germany) connected to a DC-coupled, TMS-compatible amplifier (BrainAmp, Brain Products GmbH, Gilching, Germany). Electrode placement adhered to the international 10-10 system, with the ground at AFz and the reference at FCz. Signals were digitized at 5000 Hz, and impedances were maintained below 5 kΩ throughout all recording blocks. To mask TMS-induced noise and minimize auditory evoked potentials (AEPs) [44,45,46], participants wore in-ear headphones that continuously played a custom masking sound, comprising white noise mixed with the specific time-varying frequencies of the TMS click. To further enhance masking, participants wore ear defenders (Classic Extreme Ear Defender JSP, SNR = 30 dB) over the earphones [44,47]. At the end of the recording blocks, the perceived loudness of the TMS click was evaluated using a visual analogue scale (VAS) ranging from 0 (no perception) to 10 (maximal perception).

During each experimental block, 200 TMS pulses were delivered with simultaneous EEG recording. Stimulation intensity was set to 90% of the RMT measured in that specific block (i.e., RMT_Pre-dose_, RMT_Post1_, RMT_Post2_, or RMT_Post3_). The inter-trial interval (ITI) was approximately 2 s, with a 20% random jitter.

Preprocessing of the data was performed offline by an operator blinded to the drug condition, using EEGLAB version 14.1 [48] and TESA toolboxes [49] within a MATLAB environment (Version 2021a, MathWorks, Inc., Natick, MA, USA). Trials were epoched from −1.5 to +1.5 s relative to the TMS pulse and baseline-corrected (−1.5 s to −10 ms). The data segment contaminated by the TMS artifact (−5 to 12 ms) was replaced with zeros. Following the visual rejection of trials and electrodes with prominent artifacts, a first round of independent component analysis (FastICA) was applied to isolate and remove early TMS-locked artifacts, such as cranial muscle activation and residual voltage decay [24,50]. Cubic interpolation was then applied to the previously removed artifact interval. Subsequently, the signals were downsampled to 1000 Hz, band-pass filtered (1–100 Hz), and notch filtered (48–52 Hz). Epochs were then cropped to −1 to +1 s to eliminate filter-induced edge artifacts, and a second manual rejection of single trials was performed. A second round of FastICA was conducted to detect and remove residual, non-TMS-locked artifacts (e.g., spontaneous eyeblinks and continuous muscle activity) [51]. Finally, the data were re-referenced to the common average reference.

**Figure 3:**
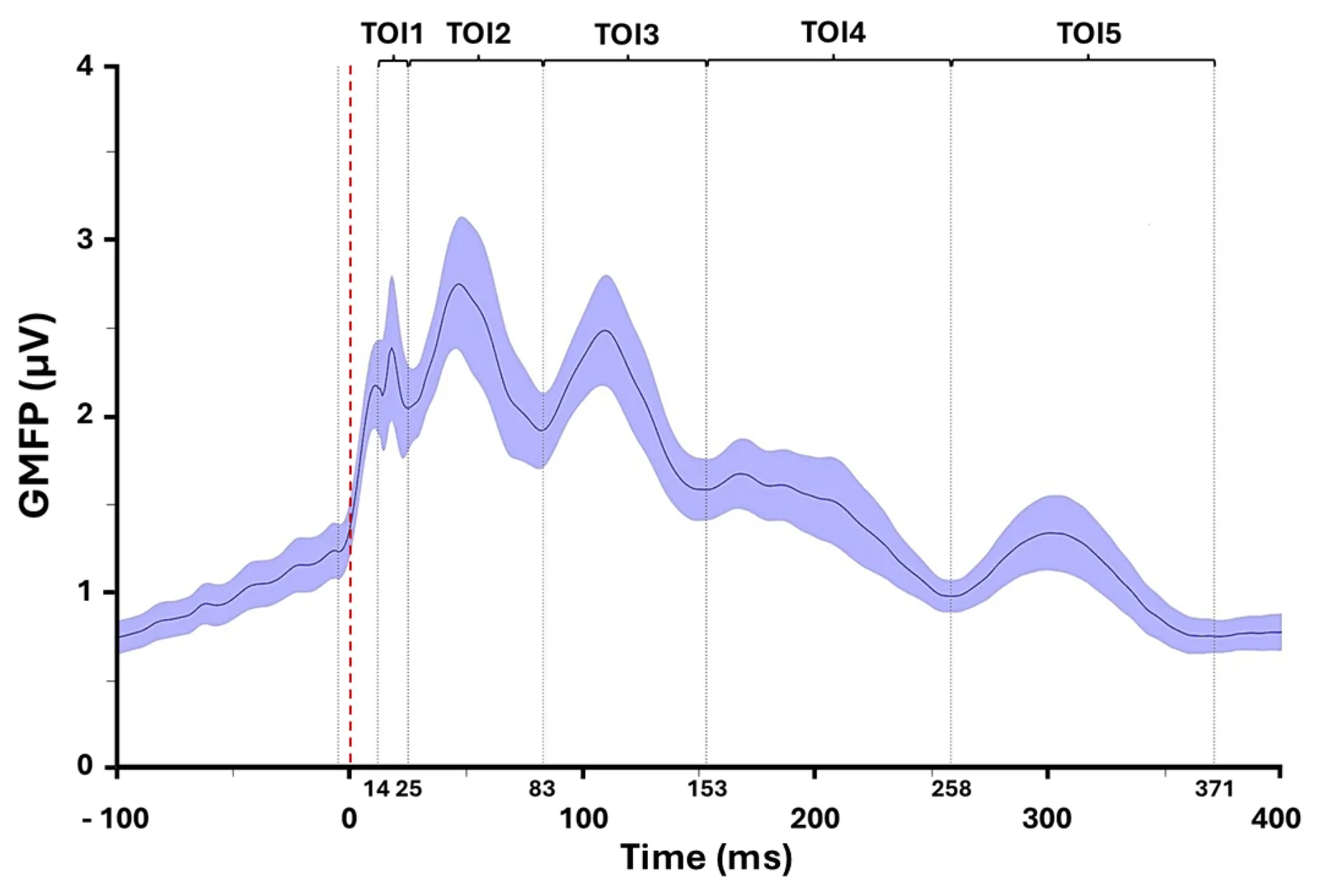
Global mean field potential. The black panel indicates the time segment of the EEG signal that was recorded around the time when the TMS pulse was cut. Vertical lines indicate the edges of TOIs. The shaded area indicate the standard error of the mean.

Time-domain analysis of the signals was based on time windows of interest (TOIs) extracted from the global mean field potential (GMFP). The GMFP was obtained by averaging data across all four sessions (Pre-dose, Post 1, Post 2, Post 3) and both experimental conditions (drug and placebo). It was computed using the standard Formula (1):

(1) $$GMFP\left(t\right)=\sqrt{\frac{\left[\sum_{i}^{k}{\left(V_{i}\left(t\right)-V_{mean}(t)\right)}^{2}\right]}{K}}$$

where *t* is time (ms), *k* is the number of channels, *V_i_* is the voltage in channel *i*, and *V_mean_* is the average voltage from all channels [44,52]. Based on the resulting GMFP troughs, five TOIs were identified: TOI1 (14–25 ms), TOI2 (25–83 ms), TOI3 (83–153 ms), TOI4 (153–258 ms), and TOI5 (258–371 ms) (Figure 3).

For time-frequency analysis, the signal was first decomposed via a Morlet wavelet transform (3.5 cycles, linear 1 Hz frequency steps from 2 to 45 Hz) using the FieldTrip toolbox (version 20220202) [53]. TRSP and inter-trial phase clustering (ITPC) were calculated according to Formulas (2) and (3), where *n* is the number of trials, *f* is the frequency, *t* is time, and *F_k_* is the spectral estimate of trial *k*:

(2) $$TRSP\left(f,t\right)=\frac{1}{n}\sum_{k=1}^{n}{\left|F_{k}\left(f,t\right)\right|}^{2}$$

(3) $$ITPC\left(f,t\right)=\frac{1}{n}\sum_{k=1}^{n}\frac{F_{k}\left(f,t\right)}{\left|F_{k}\left(f,t\right)\right|}$$

TRSP quantifies changes in power at discrete frequencies following the TMS pulse compared to a baseline segment. ITPC measures the consistency of phase angles across multiple trials, providing information about the strength of TMS-induced phase-locking [48,54]. For the TRSP, a trial-level z-transformation was applied based on the mean and standard deviation of the full-length trial. This was followed by a baseline correction for each trial, achieved by subtracting the average of the 1000 to 50 ms pre-stimulus period for each frequency [26].

### 2.5. Statistics

Motor threshold parameters (RMT, AMT), TS, and SICI were analyzed using three two-way repeated-measures ANOVAs (RM-ANOVAs), with condition (drug, placebo) and time (Pre-dose, Post 1, Post 2, Post 3) as within-subject factors. When the assumption of sphericity was violated (Mauchly’s test *p* < 0.05), the Greenhouse–Geisser correction was applied. Post hoc comparisons were conducted using paired *t*-tests or, for non-normally distributed data (assessed via the Kolmogorov–Smirnov test), appropriate non-parametric alternatives. A Bonferroni correction was applied to all main effects, interactions, and post hoc comparisons. Statistical significance was set at *p* < 0.05. All TMS-EMG statistical analyses were performed using SPSS version 22 (IBM Corp., Armonk, NY, USA).

**Figure 4:**
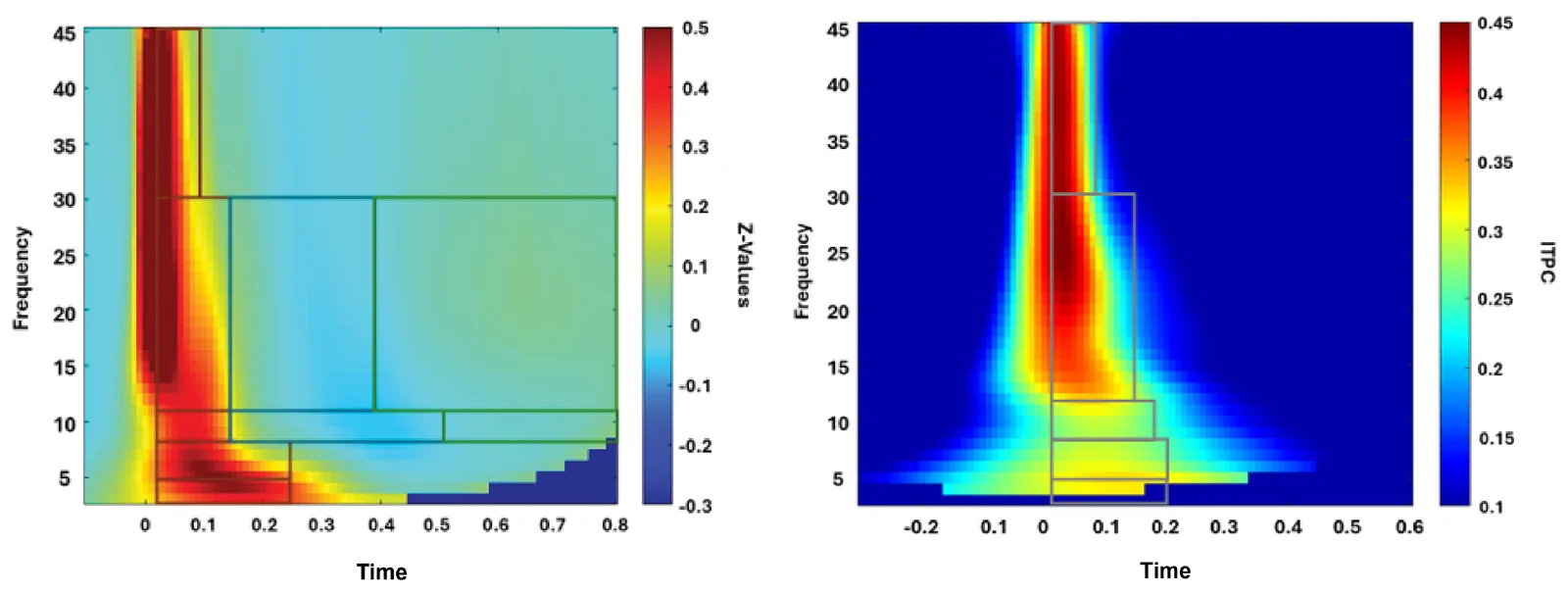
For illustrative purposes, the time-frequency representation of TRSP is shown averaged over all EEG channels for both conditions (i.e., Drug and Placebo) across all sessions (Pre, Post 1, Post 2 and Post 3). This global, condition-blind average was used to establish unbiased temporal and spectral boundaries for the subsequent electrode-level statistical analyses. The grey boxes correspond to the time and frequency windows that were used for statistical comparison. TOIs were identified as an early TOI1 for delta, theta (12–200 ms), alpha, beta (12–150 ms) and gamma (12–100 ms); a middle TOI2 for alpha (150–400 ms) and beta (150–500 ms); and a late TOI3 (500–800 ms) for alpha and beta. Right: Similarly, the time-frequency representation of ITPC is shown averaged over all EEG channels for both conditions (i.e., Drug and Placebo) across all sessions (Pre, Post 1, Post 2 and Post 3). The grey boxes correspond to the time and frequency windows that were used for electrode-level statistical comparison. TOIs were identified as: 12–180 ms for delta and theta; 12–180 ms for alpha; 12–150 ms for beta; 12–100 ms for gamma.

The analysis of TEPs, TRSP, and ITPC also utilized two-way RM-ANOVAs with condition and time as within-subject factors. Post hoc comparisons were performed using permutation testing combined with cluster-based correction for multiple comparisons. TRSP and ITPC values were averaged within specific frequency bands of interest—delta (2–4 Hz), theta (4–8 Hz), alpha (8–12 Hz), beta (13–30 Hz), and gamma (30–45 Hz)—and across previously defined TOIs derived from the GMFP. To preserve the signal-to-noise ratio, TOIs were tailored to specific frequency bands rather than applying a uniform temporal window across all frequencies. Lacking standardized criteria, these band-specific TOIs were established through an exploratory approach informed by previous literature [18,25,55,56] (Figure 4). For TRSP, the defined TOIs were: early TOI1 (12–200 ms for delta-theta; 12–150 ms for alpha and beta; 12–100 ms for gamma), middle TOI2 (150–400 ms for alpha; 150–500 ms for beta), and late TOI3 (500–800 ms for alpha and beta) (Figure 4). For ITPC, the selected TOIs were: 12–180 ms for delta, theta, and alpha; 12–150 ms for beta; and 12–100 ms for gamma (Figure 4). For ITPC, the selected TOIs were: 12–180 ms for delta, theta, and alpha; 12–150 ms for beta; and 12–100 ms for gamma (Figure 4).

To identify significant spatiotemporal clusters (channel × time) within each TOI, Monte Carlo permutation analyses (1500 iterations) were performed using dependent-sample t-statistics and multivariate ANOVAs. Cluster-based correction for multiple comparisons was implemented via the FieldTrip toolbox [53]. The minimal cluster size was set to two neighboring electrodes and a time bin of 1 ms. A cluster was defined as a set of contiguous channel-time points exceeding a preliminary threshold of *p* < 0.05. Cluster-level statistics were calculated by summing the t-values within each cluster, and significance was evaluated against a permutation-derived distribution of maximum summed t-values. A critical threshold of *p* < 0.05 was applied to all comparisons.

Finally, analyses at C_max_ for both TMS-EMG and TMS-EEG metrics were conducted using an identical statistical pipeline. Specifically, we compared the pre-dose time point against the post-dose time point corresponding to the maximum serum concentration in the drug condition, mapping this against the temporally matched data points in the placebo condition.

To explicitly model the longitudinal nature of the protocol and account for baseline variance, a secondary sensitivity analysis was conducted using linear mixed-effects models (LMMs). For TEPs, TRSP, and ITPC, the models included Condition (Drug, Placebo), Time (Post 1, Post 2, Post 3), and their interaction as fixed effects, with the corresponding baseline (Pre-dose) value as a covariate and Subject as a random intercept. Identical TOIs and frequency bands were utilized. Cluster-based permutation testing (1500 permutations, cluster-level alpha = 0.05) was applied to correct for multiple comparisons across electrodes and time.

## 3. Results

### 3.1. Participants

Of the eighteen enrolled participants, two were excluded after testing positive for nicotine at their second dosing visit, and one withdrew following the first visit. The remaining fifteen participants completed the full experimental protocol. Demographic characteristics and GWP42003-P serum concentrations are summarized in Table 1. The drug was detectable in the serum of all participants from four hours post-dose onward. No serious adverse events (SAEs) were reported during the study.

### 3.2. Effects of GWP42003-P on TMS-EMG Measures

**Figure 5:**
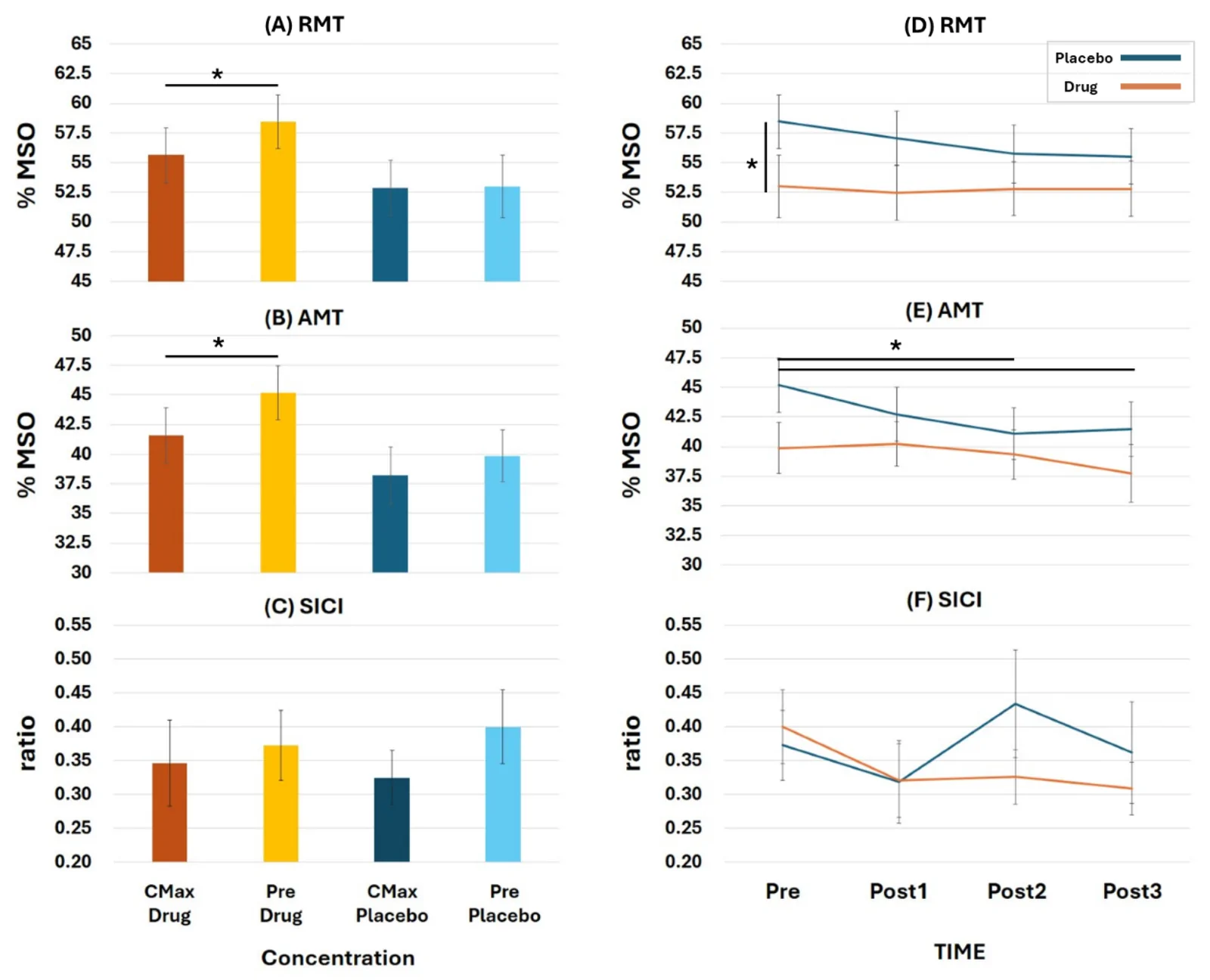
The results of RMT, AMT, and SICI are shown. The left panel illustrates the comparison between Pre-dose and Cmax for RMT (**A**), AMT (**B**), and SICI (**C**). The right panel shows the time-series changes in RMT (**D**), AMT (**E**), and SICI (**F**). Asterisk (*) denotes significance at *p* < 0.05 in the post hoc comparisons.

The two-way RM-ANOVA on RMT values revealed a significant main effect of Condition (F_1,14_ = 5.544, *p* = 0.034, $\eta_{p}^{2}$ = 0.09), with no significant Condition × Time interaction. Post hoc comparisons showed a significantly higher baseline RMT in the Placebo compared to Drug condition (*p* = 0.002, Cohen’s *d* = 0.57) (Figure 5D). For AMT, the analysis demonstrated a significant main effect of Time (F_3,42_ = 6.061, *p* = 0.002, $\eta_{p}^{2}$ = 0.12). Within the drug condition, uncorrected post hoc comparisons showed differences between Pre-dose and Post 1 (*p* = 0.018), Post 2 (*p* = 0.001), and Post 3 (*p* = 0.003), as well as between Post 1 and Post 2 (*p* = 0.020). However, following Bonferroni correction, only the differences between Pre-dose and both Post 2 (*p* = 0.006, Cohen’s *d* = 0.51) and Post 3 (*p* = 0.018, Cohen’s *d* = 0.59) remained significant (Figure 5E). No significant changes were observed in the placebo condition.

To verify TS stability in SICI across sessions and conditions, a two-way RM-ANOVA was conducted, revealing a significant main effect of Time within the placebo condition (F_3,12_ = 6.128, *p* = 0.009, $\eta_{p}^{2}$ = 0.15). While initial uncorrected paired *t*-tests suggested TS increased over time—specifically comparing Post 3 to Pre-dose (*p* = 0.005), Post 1 to Pre-dose (*p* = 0.005), and Post 3 to Post 2 (*p* = 0.043)—none of these comparisons survived Bonferroni correction. Furthermore, the analysis of SICI values showed neither a significant main effect of Condition nor a Condition × Time interaction (Figure 5F).

When examining RMT values specifically at Cmax, the two-way RM-ANOVA indicated significant main effects of both Time (F_1,14_ = 6.95, *p* = 0.020, $\eta_{p}^{2}$ = 0.18) and Condition (F_1,14_ = 5.693, *p* = 0.032, $\eta_{p}^{2}$ = 0.17). Paired *t*-tests confirmed a significant decrease in RMT at C_max_ within the drug condition (*p* = 0.020), which remained robust after Bonferroni correction (*p* = 0.025, Cohen’s *d* = 0.51) (Figure 5A). No such differences were found in the placebo condition.

Similarly, for AMT at C_max_, the analysis demonstrated significant main effects of Condition (F_1,14_ = 11.628, *p* = 0.004, $\eta_{p}^{2}$ = 0.21) and Time (F_1,14_ = 5.580, *p* = 0.033, $\eta_{p}^{2}$ = 0.22), with no significant Condition × Time interaction. Paired *t*-tests revealed a significant decrease in AMT at C_max_ within the drug condition (*p* = 0.005, Bonferroni-corrected *p* = 0.010, Cohen’s *d* = 0.95), whereas the placebo condition remained stable (Figure 5B). Consistent with the overall time-course analysis, evaluating SICI values at Cmax yielded no significant main effects or interactions (Figure 5C).

### 3.3. Effects of GWP42003-P on TMS-EEG Evoked Potentials and TMS-Related Spectral Perturbation and Intertrial Phase Clustering

**Figure 6:**
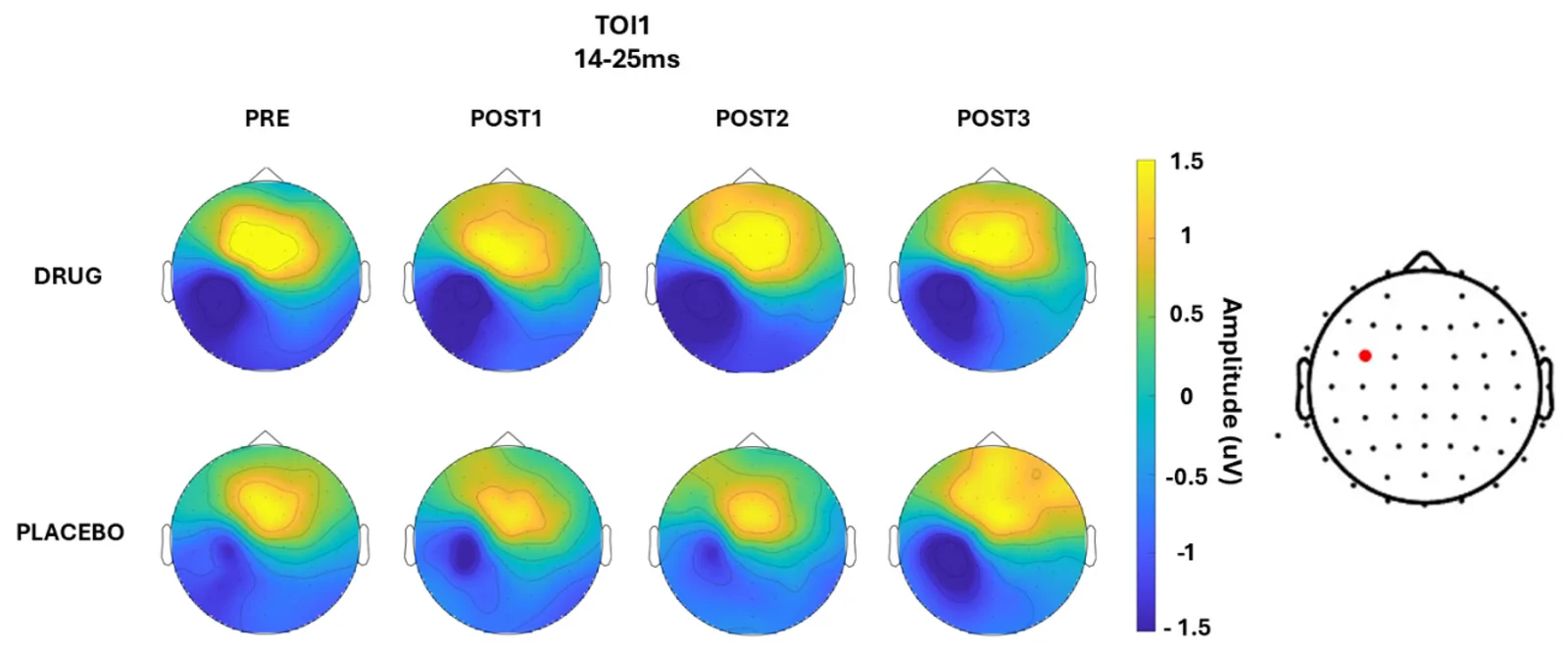
Topographical maps of TEPs between 14 and 25 ms for both conditions (i.e., drug and placebo) across sessions (i.e., Pre, Post 1, Post 2, Post 3). The highlighted electrode (red) indicates a significant effect in the ANOVA.

The two-way RM-ANOVA performed on TEPs demonstrated a significant main effect of Time in TOI1 (14–25 ms, F_3,42_ = 2.98, *p* = 0.04 $\eta_{p}^{2}$ = 0.07) at a single electrode (FC3), with no significant main effects of Condition and no Condition × Time interaction (Figure 6). Consistent with the absence of a condition-driven or interaction effect, subsequent post hoc comparisons between the drug and placebo conditions at each individual time point within TOI1 revealed no significant differences (*p* > 0.05).

**Figure 7:**
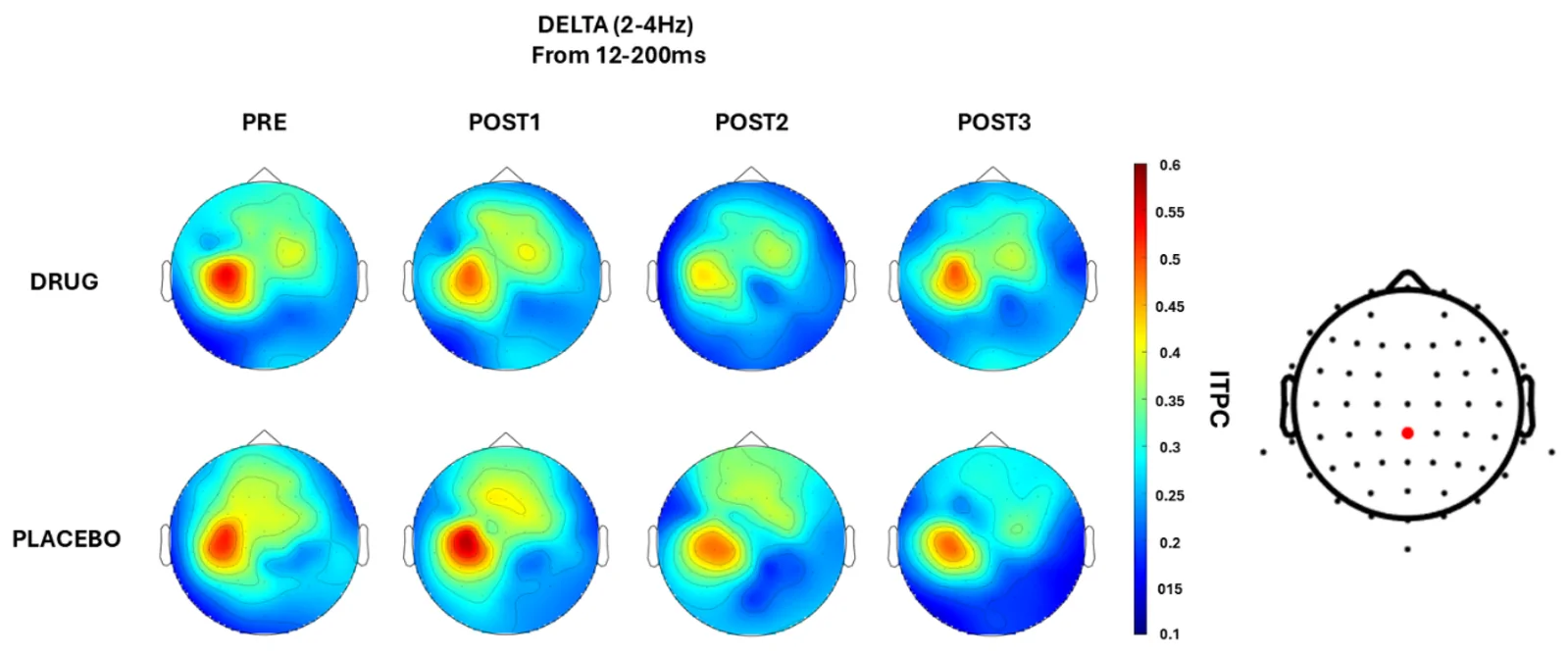
Topographical maps of delta (2–4 Hz) ITPC between 12 and 200 ms for both conditions (i.e., drug and placebo) across sessions (i.e., Pre, Post 1, Post 2, Post 3). The highlighted electrode (red) indicates a significant effect in the ANOVA.

**Figure 8:**
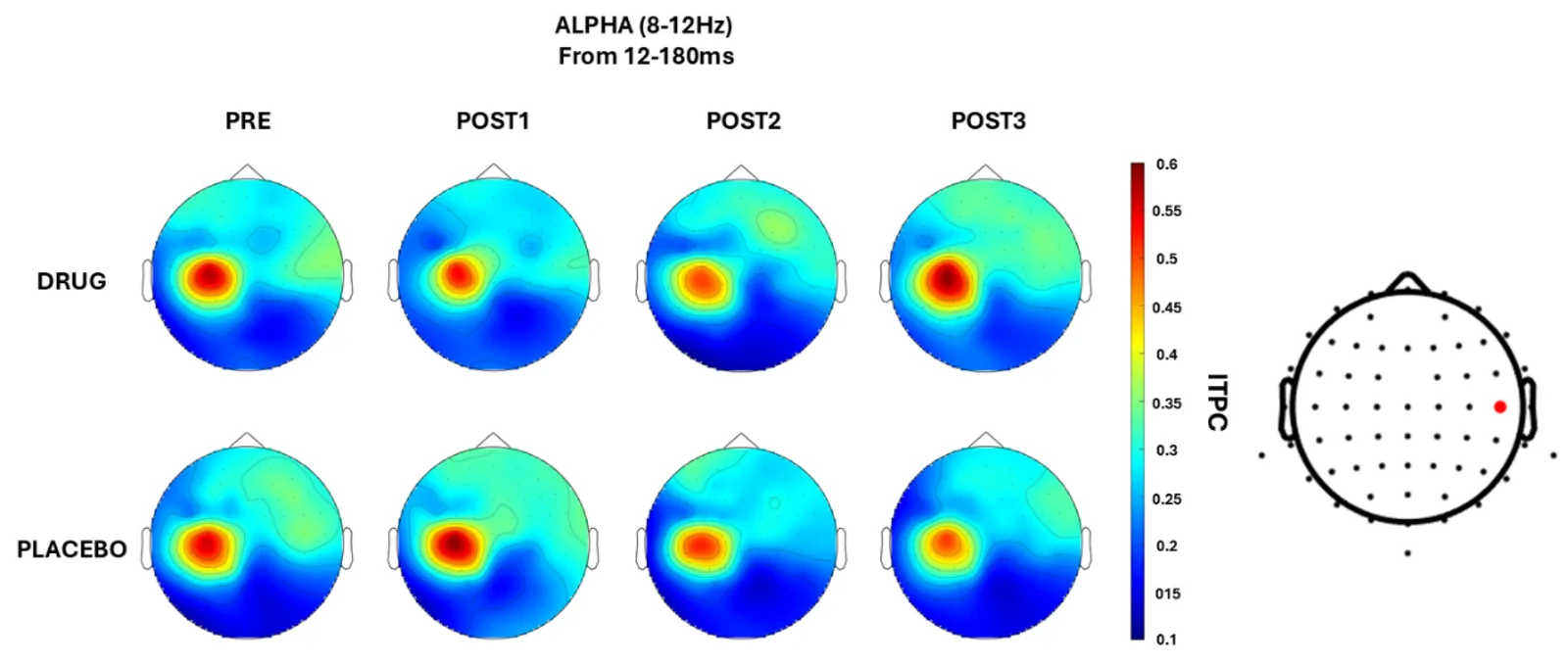
Topographical maps of alpha (8–12 Hz) ITPC between 12 and 180 ms for both conditions (i.e., drug and placebo) across sessions (i.e., Pre, Post 1, Post 2, Post 3). The highlighted electrode (red) indicates a significant effect in the ANOVA.

No significant effects were observed for TRSP. However, the analysis of ITPC revealed a significant main effect of Condition in TOI1 of the delta frequency band (12–180 ms, F_1,14_ = 5.32, *p* = 0.04, $\eta_{p}^{2}$ = 0.09), characterized by reduced ITPC in the drug condition compared to placebo at electrode CPz, indicating decreased phase-locked neural synchronization. Although these condition-driven changes appeared qualitatively more pronounced at later time points (Figure 7), they were not supported by a significant main effect of Time, a Condition × Time interaction, or individual post hoc comparisons. Similarly, a significant main effect of Condition was observed in the alpha band ITPC TOI1 (12–200 ms, F_1,14_ = 5.65, *p* = 0.03 $\eta_{p}^{2}$ = 0.1), characterized by increased ITPC in the drug condition over electrode CP6, reflecting enhanced phase-locked neural synchronization (Figure 8). Again, while these differences were qualitatively visible at later time points, there was no significant main effect of Time, no significant interaction, and no significant differences in the post hoc comparisons. All other baseline comparisons, as well as the targeted analyses performed at Cmax, were statistically non-significant (*p* > 0.05).

### 3.4. Secondary Longitudinal Modeling of TMS-EEG Metrics

**Figure 9:**
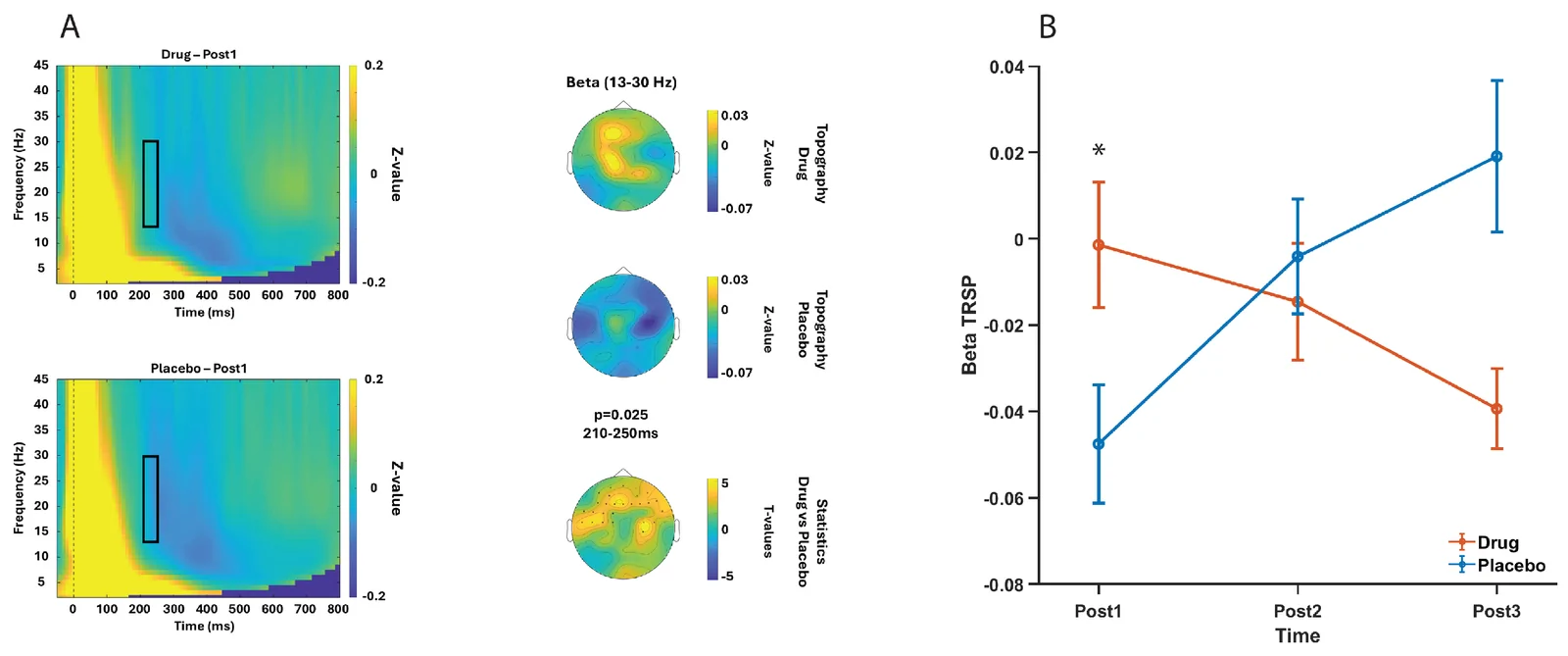
Panel (**A**): Time-frequency representations of TRSP for the Drug (**top**) and Placebo (**bottom**) conditions at the Post 1 assessment. Spectrograms reflect the average signal across the significant electrodes identified in the post hoc cluster-based permutation analysis. The black rectangle highlights the significant positive cluster in the beta band (13–30 Hz) between 210 and 250 ms. To the right, scalp topographies display the spatial distribution of beta TRSP for both conditions within this time window, alongside the statistical map (z-values) illustrating the significant difference (*p* = 0.025) over bilateral fronto-temporal regions. Panel (**B**): Longitudinal trajectory of the mean beta TRSP extracted from the significant electrode cluster across all post-dose time points (Post 1, Post 2, and Post 3). The asterisk denotes the statistically significant difference between the Drug and Placebo conditions at Post 1, following the linear mixed-effects model analysis. Error bars represent the standard error of the mean.

The secondary LMM analysis, incorporating baseline values as a covariate, yielded results largely consistent with the primary RM-ANOVA. No significant main effects or interactions were observed for TEPs or ITPC within any of the predefined TOIs (all *p* > 0.05). For TRSP, however, the LMM revealed a significant main effect of Time for beta band power (150–500 ms; F_2,69.51_ = 9.33, *p* = 0.006) and a significant Condition × Time interaction (F_2,69.60_ = 9.64, *p* = 0.045) between 180 and 250 ms. Post hoc cluster-based permutation testing revealed a significant positive cluster at Post 1 (*p* = 0.025), showing higher beta TRSP (i.e., reduced beta desynchronization) in the Drug condition compared to Placebo. No significant differences were observed at Post 2 or Post 3 (Figure 9).

## 4. Discussion

### 4.1. TMS-EEG and Cortical Synchronization

The present study explored the acute effects of GWP42003-P on cortical excitability using TMS-EEG and TMS-EMG. Pharmacokinetic (PK) data indicated that in most participants, serum concentrations of the compound became detectable after Post 2 and peaked around Post 3. Overall, our primary whole-brain analyses yielded predominantly null findings; we found no statistically significant changes across the selected TOIs for TEPs, TRSP, and ITPC. However, a qualitative, non-significant trend emerged in the drug condition compared to placebo, characterized by an increase in alpha ITPC (12–150 ms) and a decrease in delta ITPC (12–200 ms), specifically during the Post 2 and Post 3 sessions (Figure 7 and Figure 8). Because ITPC provides a measure of phase-locked neural synchronization, these trends suggest a possible, though statistically unsupported, modulation of neural responses by CBD. Consistently, ASMs known to reduce neuronal excitation, such as lamotrigine, have been shown to induce a diffuse decrease in slow oscillatory activity in patients with epilepsy [57]. Although preliminary, the trend toward decreased delta ITPC following GWP42003-P administration loosely resembles electroencephalographic patterns reported with lamotrigine in epilepsy and levetiracetam in Alzheimer’s disease [58], pointing toward a potential modulation of slow oscillatory rhythms by CBD.

GABAergic feedback from interneurons is critical to the physiological mechanisms underlying alpha rhythm generation [59,60]. These interneurons drive neural rhythms across various temporal scales. For instance, the alpha activity model proposed by Jones and coworkers highlights the necessary role of low-threshold T-type voltage-gated calcium channels, which produce currents with sufficiently slow dynamics to support alpha rhythms. Furthermore, a specific subset of somatostatin-expressing inhibitory interneurons in layer 2/3 of the mouse somatosensory cortex has been shown to generate sustained oscillations in the 3–10 Hz range [61]. Preclinical work by Henley [62] demonstrated that CBD increases GABAergic inhibition onto rat medial entorhinal principal cells—an effect blocked by both GABAA and NMDA receptor antagonists, suggesting CBD interacts with both receptor types. CBD also exhibited an additive effect when co-administered with low doses of benzodiazepine and barbiturate agonists, alongside a ceiling effect at higher doses, which is strongly indicative of positive allosteric modulation at the GABAA receptor. Similar mechanisms have been observed in human tissue cells. However, while our observed non-significant trend toward increased alpha synchronization could theoretically align with these preclinical GABAergic mechanisms, this interpretation remains speculative. The explicit absence of significant condition-driven effects in our complementary neurophysiological measures—specifically TEPs, TRSP, and SICI, which serve as robust, established in vivo markers of GABAergic inhibition—necessitates a highly cautious interpretation of these ITPC trends.

Alterations in GABAergic neurotransmission are central to several neurological disorders, including epilepsy. Although its precise mechanisms of action are not fully elucidated, CBD possesses well-documented anticonvulsant properties in preclinical models and proven anti-seizure efficacy in clinical trials for intractable childhood-onset epilepsies, such as Dravet and Lennox–Gastaut syndrome [62]. The modulatory effect of CBD on GABA receptors is broadly supported by in vitro and ex vivo studies [63]. Bakas and colleagues [64] showed that CBD acts as a positive allosteric modulator at multiple GABAA receptor subtypes, particularly those containing α2, β2, and γ2 subunits. Using human epileptic brain tissue, Ruffolo and coworkers [65] further confirmed CBD’s ability to modulate GABAA receptor function. Collectively, these findings suggest that CBD’s clinical efficacy in reducing seizure frequency and severity [66] is at least partially mediated by GABAA receptor modulation. Nevertheless, our acute in vivo human data did not yield statistically significant evidence to firmly corroborate these preclinical mechanisms at the cortical network level via traditional statistical pipelines.

Interestingly, while our primary analyses yielded largely null results, a secondary longitudinal model incorporating baseline variance revealed a localized Condition × Time interaction in the beta band. Specifically, at peak plasma concentration (Post 1), GWP42003-P administration was associated with a trend toward reduced beta desynchronization following TMS. While classical direct GABAA positive allosteric modulators (such as benzodiazepines) typically enhance beta-band reactivity and rebound, CBD exhibits a highly complex, indirect pharmacological profile (e.g., FAAH inhibition, 5-HT1A agonism, TRPV1 modulation). The transient blunting of beta desynchronization observed here may reflect a generalized dampening of cortical reactivity mediated through these non-classical pathways, rather than direct GABAergic transmission. However, given that this effect did not survive strict multiple comparison corrections in the post hoc phase, it must be interpreted with caution and viewed primarily as a potential pharmacokinetic biomarker for future, larger-cohort investigations.

### 4.2. TMS-EMG and Motor Excitability

Regarding TMS-EMG measures, we observed baseline differences in RMT between the drug and placebo conditions, alongside a progressive decrease in AMT within the drug condition at Post 2 and Post 3. Conversely, motor thresholds in the placebo group remained stable over time. Motor thresholds are thought to reflect the axonal excitability of cortico-cortical fibers, their excitatory synaptic contacts, and the initial axon segments of corticospinal neurons. While ASMs that block voltage-gated sodium channels—such as carbamazepine [10,67,68,69], lacosamide [10,11] and lamotrigine [69,70,71]—typically increase motor thresholds, drugs modulating CNS neurotransmitters (e.g., GABA, dopamine, noradrenaline, serotonin, acetylcholine) yield inconsistent effects on these parameters [72].

The observed pre-drug baseline difference in motor thresholds contrasts with previous literature establishing good test–retest reliability for this metric [73]. However, this discrepancy could be partially attributed to daily fluctuations in individual neurophysiology driven by factors such as sleep architecture [74] and diet [75]. Indeed, Cotovio and colleagues [76] demonstrated that RMT varies by more than 5% in nearly half of all repeated testing sessions when compared to baseline measurements.

### 4.3. Interpretation of the Null Findings

The predominantly negative nature of our primary neurophysiological findings is a critical aspect of this study. The absence of statistically significant widespread results aligns with prior research indicating that CBD alone does not significantly alter TEPs obtained with single-pulse TMS [28] or resting cortical oscillations [77]. While post hoc analyses revealed a decrease in RMT and AMT over time within the drug condition, the lack of a significant Condition × Time interaction precludes a definitive attribution of these changes to GWP42003-P. Moreover, the lack of significant condition-specific differences across SICI, TEPs, and the primary TRSP analysis firmly suggests that a single acute dose of CBD does not induce widespread, robust alterations in global cortical excitability. While qualitative trends in neural synchronization were observed alongside a localized, non-classical beta-band modulation, the lack of corroborating effects in established primary biomarkers dictates that preclinical evidence of CBD’s GABAergic modulation does not readily translate to acute cortical responses in a healthy male population. As suggested by recent TMS-EEG investigations, a single acute dose of CBD may simply be insufficient to produce robust systemic alterations, whereas chronic administration might be required to exert stronger and more consistent modulatory effects [28].

### 4.4. Pharmacokinetic Considerations

Our PK data present a notable limitation. While steady-state CBD typically reaches $C_{max}$ at approximately 3 h (range: 2.5–5.0 h) following twice-daily dosing of 750 mg or 1500 mg, seven participants (43.75%) in our acute dosing paradigm reached their peak serum concentration at 6 h post-dose (Post 3) (Table 1). It is important to note that this delayed absorption is unlikely to be driven by a ‘food effect’. Previous pharmacokinetic studies demonstrate that high-fat meals significantly increase the rate and extent of CBD absorption; therefore, our participants were administered a standardized, low-fat meal (300 kcal, 4 g fat) specifically to prevent these absorption spikes and limit inter-individual variability. Because no assessments were conducted beyond the 6 h mark, several participants may not have achieved their true $C_{max}$ during the observation window. This truncation likely impacted our ability to capture the maximum timing and intensity of CBD’s cortical effects.

## 5. Conclusions and Limitations

In this Phase 1 clinical trial, we employed a multimodal neurophysiological approach to investigate the acute effects of a single dose of GWP42003-P. The primary neurophysiological metrics did not demonstrate statistically significant condition-driven alterations, indicating that a single acute dose of highly purified CBD does not broadly modulate widespread cortical excitability or established GABAergic biomarkers. While a secondary longitudinal model revealed a localized, transient blunting of beta desynchronization at peak plasma concentration, and qualitative trends in delta and alpha synchronization were observed, these findings remain preliminary and cannot be definitively attributed to the drug.

Several limitations should be considered when interpreting these results. First, the final sample size of 15 participants is relatively small, which may have limited the statistical power to detect subtle neurophysiological effects, such as the beta-band modulation. Consequently, future investigations utilizing larger sample sizes are indicated. The sample was also restricted to a cohort of healthy adult males, limiting the generalizability of the results to female populations or clinical cohorts. Furthermore, despite the small sample size, the age of participants spanned 21 years. Because of age-related changes in intrinsic cortical excitability, TMS-induced electric field distribution, and baseline EEG characteristics can introduce substantial variability in neurophysiological responses, this wide age range represents a notable limitation. Future large-scale investigations should account for this variability by utilizing age-stratified analyses or incorporating age as a covariate.

Additionally, demographic data regarding participant ethnicity were not recorded in this trial; therefore, we cannot account for potential inter-individual metabolic variability in CBD absorption and clearance driven by ethnicity-associated genetic polymorphisms. Because this study did not identify widespread significant effects of the drug on primary EEG parameters, we did not perform direct continuous statistical correlations between exact plasma CBD concentrations and neurophysiological metrics; future trials should explicitly explore these direct pharmacokinetic–pharmacodynamic relationships. Second, this study relied solely on a healthy volunteer cohort evaluated against a placebo control. The absence of clinical comparison groups—such as patients with drug-resistant epilepsy where CBD has proven clinical efficacy, or individuals currently taking other anti-seizure medications—restricts our ability to determine how underlying pathological states might alter the neuropharmacological response to CBD.

Overall, the largely null findings of the present study represent an important scientific contribution to the emerging field of CBD neuropharmacology. This absence of acute, widespread effects provides valuable baseline evidence that can guide future investigations. Future research utilizing chronic dosing regimens or clinical patient populations may be better positioned to capture distinct neuropharmacological effects, which could ultimately contribute to the development of more targeted treatment strategies across a wide range of neurological and neuropsychiatric disorders.

## Figures and Tables

**Table 1:** Demographic information and serum concentrations of GWP42003-P.

				NTIME							
				0 h	1 h	1 h 45 min	3 h	4 h	4 h 45 min	6 h	6 h 45 min
ANALYTE	SUBJID	AGE	DAY	AVAL (ng/ML)							
CBD	1001	25	2	0.00	53.8	83.4	181	335	309	141	141
	1002	29	1	0.00	163	252	265	428	554	1070	774
	1004	26	1	0.00	0.00	0.00	3.99	6.7	8.12	88.1	274
	1005	27	2	0.00	12.4	11.3	84.2	112	60.1	149	222
	1006	42	2	0.00	19.3	129	288	327	226	776	668
	1007	35	1	0.00	0.00	0	77.4	147	143	272	245
	1010	38	2	0.00	75.4	93.3	526	317	480	2090	975
	1011	29	1	0.00	96.4	297	207	462	353	230	125
	1012	27	2	0.00	12.2	57.9	116	131	130	417	577
	1013	26	1	0.00	0.00	0	106	83.6	26.2	16.2	133
	1014	22	2	0.00	160	160	129	302	272	341	171
	1015	26	1	0.00	5.10	8.51	17.5	12.7	41.4	92.4	395
	1016	34	2	0.00	0.00	0.00	94.6	259	298	541	398
	1017	39	1	0.00	0.00	5.73	13.4	14.3	14	62.6	124
	1018	40	1	0.00	0.00	0.00	6.08	358	312	966	1360
	6003	25	2	0.00	0.00	0.00	34.2	35.6	136	407	356
	6013	43	1	0.00	119	579	688	401	280	303	283
**N**				17	17	17	17	17	17	17	17
**Mean**				0.00	42.2	98.7	167	220	214	410	425
**SD**				0.00	58.5	155	189	159	162	358	346
**Min**				0.00	0.00	0.00	3.99	6.70	8.12	16.2	124
**Median**				0.00	12.2	11.3	106	259	226	303	283
**Max**				0.00	163	579	688	462	544	1090	1360
**Geometric Mean**							79.4	127	130	256	323
**Geometric CV%**							283	235	198	163	86.7

## Data Availability

The datasets presented in this article are not readily available because the data were generated during a Phase I clinical trial sponsored by Jazz Pharmaceuticals, and strict proprietary and ethical restrictions apply to their public distribution. Requests to access the datasets should be directed to the corresponding author.

## Abbreviations

AEP	Auditory evoked potential
AMT	Active motor threshold
ASM	Anti-seizure medication
BID	Twice daily (bis in die, referring to dosing frequency)
CBD	Cannabidiol
Cmax	Maximum serum/plasma concentration
CNS	Central nervous system
EEG	Electroencephalography
EMG	Electromyography
FastICA	Fast Independent Component Analysis
FDI	First dorsal interosseous (muscle)
GABA	Gamma-aminobutyric acid (GABAAR/GABABR refer to specific receptor subtypes)
GMFP	Global mean field potential
GPR55	G Protein-Coupled Receptor 55
ITI	Inter-trial interval
ITPC	Inter-trial phase clustering
M1	Primary motor cortex
MEP	Motor evoked potential
NMDA	N-methyl-D-aspartate (receptor)
PK	Pharmacokinetics
RM-ANOVA	Repeated-Measures Analysis of Variance
RMT	Resting motor threshold
SAE	Serious adverse event
SICI	Short intracortical inhibition
SNR	Signal-to-noise ratio (also used for ear defender sound attenuation rating)
TEP	TMS-evoked potential
Tmax	Time to maximum plasma concentration
TMS	Transcranial magnetic stimulation
TOI	Time window of interest
TRPV1	Transient Receptor Potential Vanilloid Type 1
TRSP	TMS-related spectral perturbations
TS	Test stimulus
VAS	Visual analogue scale
